# Seroprevalence of SARS CoV-2 among children after the second surge (June 2021) in a rural district of South India: Findings and lessons from a population-based survey

**DOI:** 10.3389/fped.2022.997684

**Published:** 2022-11-07

**Authors:** Carolin Elizabeth George, Leeberk Raja Inbaraj, Shon Rajukutty, Roshni Florina Joan, Sangeetha Muthuraj, Sindhulina Chandrasingh

**Affiliations:** ^1^ Division of Community Health and Family Medicine, Bangalore Baptist Hospital, Bangalore, Karnataka, India; ^2^ Department of Microbiology, Bangalore Baptist Hospital, Bangalore, Karnataka, India

**Keywords:** COVID-19, COVID-19 in children, seroprevalance, rural India, pandemic (COVID19)

## Abstract

**Objective:**

To determine the seroprevalence of SARS COV 2 among children in the Bangalore Rural district.

**Methods:**

We conducted a cross-sectional study after the second surge of COVID-19 from 14 June to 13 July 2021 and recruited 412 children through house to house visits from four villages in a rural district. We administered a questionnaire to collect demographics and details of COVID-19 infection and used the ABCHEK Antibody Card test (NuLifecare,India) which is an ICMR approved test for detecting antibodies (IgG & IgM) by immunochromatography using the finger prick method. We used Statistical Package for the Social Sciences version 20.0 for analysis.

**Results:**

Our participants had an age group ranging from 11 months to 18 years. There was an almost equal distribution of boys (48.3%) and girls (51.7%). We estimated seroprevalence of 45.9% (95% CI: 41–50.8) among children. Seroprevalence was significantly associated with a history of symptoms suggestive of COVID-19 in the past, the seropositive status of the parents, and any other family members being tested positive. Age and gender of the child, education and occupation of the parents were not associated with the seropositivity status of the child.

**Conclusion:**

Seroprevalence of COVID-19 among children corresponds to adult seroprevalence during the same time interval. This knowledge can be of practical application where adult prevalence is documented. Unvaccinated children in low-resource settings need special attention with respect to monitoring for new mutations as well as managing endemic needs.

## Introduction

In India, the first case of COVID-19 was reported on 30 January 2021. As of 09 September 2022, India had documented 44 million COVID-19 cases and 0.5 million COVID-19 deaths (1). By that time, India had seen two waves, first in late 2020 and then in early April of 2021. The second pandemic wave was far more devastating because of accelerating cases and a crunch for life-saving hospital beds and supplies (2, 3). Infection in the younger population, happy hypoxia and a relatively sudden reduction in oxygen saturation, increased opportunistic infections like mucormycosis and a higher number of deaths made the second wave direr than the first wave (4, 5).

Since the second wave was graver than what the experts and the public expected, the thought of a third wave still lingered on everyone's minds even though the ICMR 4th serosurvey reported a seroprevalence of 67.6% (6). Documented high seroprevalence and vaccination among adults reduced the possibility of a third wave of high amplitude. In this context, one of the important considerations was the vulnerability of children with COVID-19 as they formed a significant proportion of the unvaccinated population. There was limited seroprevalence data among children at the time of conducting the study. Since then, outbreaks of COVID-19 have been identified in different countries in schools and day care centres. There are reports which indicate that younger children may be less infectious, as measured by secondary attack rates, than adolescents and adults (7).

Though SARS-CoV-2 is thought to cause less severe illness and fewer deaths in children and adolescents compared to adults, it’s important to note the multisystem inflammatory syndrome in children (MIS-C) reported in many countries, a severe manifestation of the virus. Although it affects multiple systems, the cardiovascular signs are the most prominent. These patients exhibit high anti-SARS-CoV-2 antibody titres an it mimics Kawasaki disease (KD). Being a novel disease entity, the immunopathogenesis of this condition is not fully understood (8). In addition to being critically ill requiring ICU admissions, the mortality has been as high as 9% in children in with MIS-C (9).

Children and adolescents also remain susceptible to the infection and form a part of the transmission chain. The risk of both infection and transmission is reported to increase with age. As reported to the WHO, during the initial pandemic phase with the ancestral strain during the time period from 30 December 2019 to 25 October 2021, children under five years of age represented 2% of reported global cases. Older children (5 to 14 years) accounted for 7% and older adolescents and young adults (15 to 24 years) represented 15% of reported global cases (10, 11). Persistence of anti–SARS-CoV-2 spike receptor-binding domain IgG was seen in a household cohort study in Italy even following asymptomatic infections until 12 months after infection in all age groups and higher levels of binding antibodies were seen in children younger than 3 years when compared with adults older than 18 years. In addition to direct benefits, vaccinating children would be expected to decrease transmission in this age group and also transmission from children and adolescents to older adults. COVID-19 (12).

The Community Health Division (CHD) of Bangalore Baptist Hospital has been providing curative and preventive health services through a Rural Health Centre and network of mobile clinics to residents of Bangalore rural district for over a decade. Though these rural areas were spared in the first wave (seroprevalence 12%), the virus swept through the rural landscapes, affecting most of the population during the second surge (13). A serosurvey in June 2021 reported a prevalence of 63.2% among adults in Bangalore rural district (14). When the report was communicated to the community in the surveyed area, the community was interested in knowing whether their children were infected during the delta surge. Hence we designed a study with the objective to determine the seroprevalence of COVID-19 among children in the Bangalore Rural district. The result of the study is of interest to people in the communities and local authorities for resource planning purposes. Our findings will be of research and policy importance as there are limited publications regarding COVID-19 transmission and seroprevalence among children from rural India.

## Methodology

We conducted a cross-sectional study based on WHO's recommendations in the Bangalore rural district. A detailed description of this district, including population, occupation and health care services provided, can be found in our earlier publication (13). We randomly selected one of the four sub-districts and then randomly chose four villages from the selected sub-district. Based on our earlier adult survey, we assumed a seroprevalence of 30%, with a relative precision of 10% and a design effect of 1.2; the sample size was calculated as 403. The study was approved by the Ethics committee of Bangalore Baptist Hospital on 23 June 2021.

We obtained parental consent for children under 14 years and parental consent and verbal assent from the participants between 14 and 18 years. We recruited all the eligible children (0–18 years) through a house-to-house survey from 14 June to 13 July 2021 after the required consent/assent. Children who were seriously ill with any other chronic conditions or had any established comorbidities were excluded. We administered a questionnaire on demographics (age, gender, education of the parents) and the history of COVID-19 infection among the participants and family members. The interviewer used Epi-info 7.0 TM mobile application-based tool to record replies offline, which were later downloaded for analysis. We used an ICMR approved point of care card test, ABCHEK Antibody Card [NuLifecare, Noida (UP), India], for detecting IgG and IgM antibodies by immunochromatography using the finger prick method (15). We counselled the parents and children and reassured them in order to reduce the anxiety and pain due to needle prick before collecting the sample. The test has a total co-incident rate of 92.8% and 96.5% for IgM and IgG antibodies, respectively, as per the manufacturer (15).

This test kit was evaluated in our laboratory using the Elecsys SARS CoV-2-S assay which tests for total antibodies including IgM and IgG (Roche Diagnostics). The measurement range of the assay is from 0.40 U/ml to 250 U/ml. Levels of <0.80 and ≥0.80 U/ml were considered as negative and positive respectively according to the manufacturer’s recommendations (16). The evaluation was done with a total of 30 samples of known serostatus. All the seronegative samples and samples with total antibody levels greater than 160 U/ml gave concordant results. The card test could not detect antibody levels less than 160 U/ml.

We used Statistical Package for the Social Sciences version 20.0 for analysis. The unadjusted COVID-19 IgG antibody's seroprevalence was reported in percentage with a 95% confidence interval (CI). Using chi-square tests, the relationship between seroprevalence and comorbid conditions and socio-demographic characteristics were investigated.

## Results

We conducted a serosurvey among 412 children with age groups ranging from eleven months to eighteen years. One-third were in the age group of 6–10 years (35.2%). There was an almost equal distribution of boys (48.3%) and girls (51.7%). The majority of the parents were educated till high school (fathers – 53.6%, mothers - 44.7%). More than two-thirds (65.5%) of the mothers were homemakers, and one-third of the fathers (35.7%) were farmers.

Twelve children (2.9%) reported having positive IgG status in the past, while 15% gave a history of at least one symptom suggestive of COVID-19 in the last month. Among the parents, 13.3% of fathers and 17.2% of mothers were detected as positive for COVID-19. Our Study population did not have children who lost their parent/s due to COVID-19. Similarly, 17.7% reported at least one family member was found to be positive in the past. We estimated a seroprevalence of 45.9% (95% CI: 41–50.8) among children in the rural district. We also found that the seroprevalence was higher in the age group of 0–5 years (Table 1).

**Table 1:** Age-wise distribution of unadjusted seroprevalence.

	Category	Male	Prevalence (95% CI)	Female	Prevalence (95% CI)	Total	Overall prevalence (95% CI)
Age (years)	0–5	44	54.5 (38.8–69.6)	39	61.5 (44.6–76.6)	83	57.8 (46.5–68.6)
	6–10	80	35.0 (24.7–46.5)	65	36.9 (25.3–49.8)	145	35.9 (28.1–44.2)
	11–15	65	43.1 (30.8–56.0)	64	57.8 (44.8–70)	129	50.4 (41.5–59.3)
	16–18	24	54.2 (32.8–74.4)	31	35.5 (19.2–54.6)	55	43.6 (30.3–57.7)
	Total	213	43.7 (36.9–50.6)	199	48.2 (41.1–55.4)	412	45.9 (41.0–50.8)

Seroprevalence was significantly associated with history of symptoms suggestive of COVID-19 in the past, the seropositive status of the parents, and any other family members being tested positive. Age and gender of the child, education and occupation of the parents were not associated with the seropositivity status of the child (Table 2).

**Table 2:** Factors associated with seropositivity among children.

Factors	Categories	Serological status		Total	*p*-value
		Reactive	Non - Reactive		
Age in years	≤10	100 (43.1)	128 (56.1)	228	0.35
	>10	89 (48.4)	95 (51.6)	184	
Gender	Male	93 (43.7)	120 (56.3)	213	0.36
	Female	96 (48.2)	103 (51.8)	199	
Education of the father	Lower (≤10 years)	132 (44.6)	164 (55.4)	296	0.40
	Higher (>10 years)	57 (49.1)	59 (50.9)	116	
Education of the mother	Lower (≤10 years)	121 (46.9)	137 (53.1)	258	0.58
	Higher (>10 years)	68 (44.2)	86 (55.8)	154	
Occupation of the father	Farmer	60 (40.8)	87 (59.2)	147	0.85
	Others	129 (48.7)	136 (51.3)	265	
Occupation of the mother	Housewife	123 (45.6)	147 (54.4)	270	0.12
	Others	66 (46.5)	76 (53.5)	142	
Father's serostatus	Reactive	32 (58.2)	23 (41.8)	55	0.04*
	NR/Not done	157 (44)	200 (56)	357	
Mother’ Sero status	Reactive	42 (59.2)	29 (40.8)	71	0.01*
	NR/Not done	147 (43.1)	194 (56.9)	341	
History suggestive of COVID–19	Yes	39 (56.5)	30 (43.5)	69	0.05*
	No	150 (43.7)	193 (56.3)	343	
Tested positive	Yes	8 (66.7)	4 (33.3)	12	0.14
	No	181 (45.2)	219 (54.8)	400	
Family members tested positive	Yes	44 (60.3)	29 (39.7)	73	0.006*
	No	145 (42.8)	194 (57.2)	339	

*significant *p*-value.

## Discussion

Our study revealed a high seroprevalence (45.8%) of COVID-19 infection among children in the Bangalore Rural district. This was lower than the seroprevalence among vaccine-naive adults in the same population during the same period (63.2%) (14). The only other study conducted during the same period among children from India quoted a 55.7% seropositivity rate (using IgG ELISA) among children and a corresponding 63.5% seropositivity in adults (17). Geographic variation (different states), the difference in assays and the time period would have resulted in a slight difference in prevalence.

Many studies on the paediatric population using different methods for serologic testing, using different antigens for SARS CoV2, (nucleocapsid and spike protein), done at hospitals, schools and communities have been published. Some of these show a lower seroprevalence from 0.23% to 14.4%, including 0.23% in Australia (November 2020 to March 2021), 10.8% in Germany (October to March 2021), 8.4% in Canada (March to April 2021) and 14.4% Belgium (September to October 2020) (18–21). Some of the studies have shown a higher seroprevalence such as 37% from Melbourne, Australia (May to October 2020), where the proportion in children was lower than that seen in adults (37% vs. 72%), 46.7% in Romania (March to June 2021), where it was seen to be similar to that in adults (45.6%) and increasing from 52.8% in January 2021 to 81.8% in September to October 2021 in Delhi, India (22–24). Since the vaccination among children was started later than adults, these studies were done prior to vaccination, however a direct comparison is not possible as they have been done around the world at different time points and relating differently to the waves of the pandemic in each country or continent except to understand that there are wide differences noted in seroprevalence, and some have shown a difference when compared to adults, while others have shown similar levels of seroprevalence.

The card test that we used did not pick up antibody titres less than 160 U/ml, which would have underestimated the seroprevalence in our study. There was limited data at the time of the study regarding the level of antibody production in children when infected (25). Distinct antibody responses in children when compared to adults might also lead to underestimation of the seroprevalence (25).

The vulnerability of children in getting an infection was a debate among many experts and communities, equally. The debates on transmission and its implications are legitimate, as the paediatric segment forms the bulk of the unvaccinated population. The absolute numbers of Covid infected children documented in the second wave were higher than in the first wave; however, the proportion of children remained more or less the same during the first and second waves (26, 27). During the second wave (15 March – 31 May 2021), 8.57% of infections were reported in children in the 11–20 year age group and 3.05% in the 1–10 year group (28). In the first wave, similar proportions of 8.03% and 3.28% were reported, respectively (28). The Lancet COVID-19 Commission India Task Force said that less than 5 per cent of children would require hospital admission in COVID-19, 5% with severe disease and out of them, the mortality is 2 per cent (29). With a high seroprevalence rate as close to adults, it is likely that the virus transmits to the paediatric population effectively however there were not many symptomatic cases requiring hospitalisation among children.

Five months post-study, in the month of January 2022 (30), an omicron wave hit India, including Bangalore rural district. A handful of new cases in early December was replaced by 100,000 new cases in early January 2022 (30). Though there was a rapid explosion of cases in a few weeks’ time, the hospitalisation caused by the third wave was meagre. The disease was milder in nature and in most cases, COVID-19 was an incidental cause than a reason for hospital admission, both among adults and children (31). Though the trajectory was steeper, the peak was smaller in magnitude than the second wave (347,000 as compared to 414,000 in the second wave) (32). India’s third wave trajectory is remarkably different from most other badly affected countries, where the peak of the Omicron wave was two to four times higher than their previous peaks. One of the logical explanations is hybrid immunity (33) - population’s (both adults and children) high exposure to the virus in the delta surge and a reasonable level of vaccination (adults) prior to the Omicron wave in January 2022 (31).

Though most children suffered from milder diseases, the health system's capability to handle serious paediatric cases is limited in most Indian settings, leave alone rural areas. Rare but severe COVID-19 cases and Multi-system Inflammatory Syndrome (MIS-C post-COVID infection) will be challenging to handle in the background of an 82% shortage of paediatricians in primary health centres (34, 35). This vulnerability demands a wake-up call to build up primary paediatric healthcare capacities in rural areas. Though the virus is causing lesser consequences with time, we must not forget the vulnerability of low-resource settings. This is evident by the fact that both new mutations, Delta and Omicron emerged in less wealthy countries (36). Unvaccinated population in low-resource setting needs special attention with respect to monitoring for new mutations as well as managing endemic needs.

Health system vulnerability forced the government to shut the schools for almost one and a half years, creating a privilege gap. The price of the good intention of saving lives is a widened learning gap and an aggravated socio-economic divide (37, 38). Children from poor households have forgotten to read and write, many are redeployed to work, and others are married because of the stark digital divide (39, 40). This effect fell disproportionately on girls and children belonging to lower castes. It is important to recognise the learning loss and put in structures to bridge the learning gap. Another important strategy is to come up with practical plans for continuing education keeping in mind the future local spike and sporadic outbreaks. Freezing to immobility by closing corridors of learning may not be a wise response to endemic COVID-19 infections.

Vaccination among children is also an essential topic of discussion as vaccine trials are progressing among children worldwide. In India, an expert panel of the Drug Control General of India has recommended granting emergency use authorization (EUA) to Bharat Biotech's Covaxin for children aged two to eighteen years with certain conditions (41). In India, COVID-19 vaccines are available for children over the age of 12 years since January 2022. As on 09 September 2022, 80 million children between 12 and 18 years of age have been administered two doses of vaccine (1). Vaccinating children has not resulted in any major adverse reactions in untoward incidents in India thus far. The advantages of vaccinating children has several indirect benefits apart from direct health benefits. It has given them access to education in physical class rooms, enhanced overall well being and reduced the anxiety of the parents. It has also helped reduced the transmission from children to adults.

The study has many strengths. Firstly, this is one of the very few COVID-19 seroprevalence studies among children from rural India, a population often underrepresented in research. Secondly, the study has a good sampling strategy (multi-stage random sampling and household visits) and an adequate sample size. Thirdly, having estimated the adult seroprevalence in the same district simultaneously sheds light on the transmissibility of COVID-19 in the paediatric population in relation to adult infection (14). The relationship between adult and child seroprevalence is valuable in making assumptions seroprevalence of one group is known. Though it is a valuable study, the study has a few limitations. The antibody card test would have underestimated the seroprevalence. However, we do not know how much it would have underestimated the true seroprevalence. Another limitation is that the findings of the study are to be extrapolated keeping in mind that the external validity is limited as it is conducted in a single rural district of Karnataka.

## Conclusion

During the second surge, the study in a rural district of South India showed a high seroprevalence of COVID-19 infection among children (aged less than 18 years). It concludes that the seroprevalence of COVID-19 among children corresponds to adult seroprevalence during the same time interval. This knowledge can be of practical application where adult prevalence is documented. Though mild, the rural primary health setting should be oriented to diagnose and manage children with rare but severe Covid 19 cases and MIS-C. Considering the equity issues, vaccination among children in low-resource settings is paramount as it is the primary defence against severe disease and future mutations. Further investigation is needed to evaluate the effectiveness of vaccines and to assess the immunological response in children. The study also emphasises the need for local plans to ensure continued education and social interaction in children as future local spikes are inevitable.

## Data Availability

The raw data supporting the conclusions of this article will be made available by the authors, without undue reservation.
